# Where is the patient’s chair? Differences in general practitioner consultation room layouts - an exploratory questionnaire

**DOI:** 10.12688/f1000research.19565.2

**Published:** 2020-04-17

**Authors:** Mayara Floss, Kyle Hoedebecke, Josep Vidal-Alaball

**Affiliations:** 1 Grupo Hospitalar Conceição, Porto Alegre, Brazil; 2 Family Medicine, Uniformed Services University of the Health Sciences, Bethesda, MD, USA; 3 Health Promotion in Rural Areas Research Group, Institut Català de la Salut, Sant Fruitós de Bages, Spain; 4 Unitat de Suport a la Recerca de la Catalunya Central, Fundació Institut Universitari per a la recerca a l'Atenció Primària de Salut Jordi Gol i Gurina, Barcelona, Spain

**Keywords:** primary health care, health facilities, facility design and construction, doctor-patient relations, health communication, family medicine

## Abstract

Background: Consultation room design varies from country to country. The layout of a general practitioner’s (GP’s) consulting room may influence the physician’s or patient’s experience. The aim of this study is to explore and investigate the layout of GP’s consulting rooms around the world and to describe any significant differences.

Methods: Between 3rd July and 2nd August 2018, an internet-based questionnaire on Google Docs was distributed by email, social media and WhatsApp platforms to several worldwide rural medicine groups.  Analysis of an internet-based questionnaire to explore possible layouts of consultation rooms within practices was performed. The questionnaire was designed with three distinct sections: first, a GP demographic profile including gender, year of graduation from medical school, country of graduation, and type of practice (private or public); second, questions relating to the office layout; third, a section for questionnaire feedback.

Results: 502 responses to the questionnaire were received; 65.3% women and 34.7% men.  The most common layout in Europe and America was where the physician and the patient were separated by a desk. The layout where the physician and the patient had a 90º angle facing each other was the most commonly used layout in Asia-Pacific and Africa. For GPs who graduated before 1990 and between 1990-2010, the layout where the table was between the patient and physician was preferred. However, physicians graduating after 2010 preferred a layout with the physician and the patient with a 90º angle facing each other.

Conclusion: The position of the GP’s desk differs between and within countries as well as the gender of the physician and year of graduation. Next steps should focus on gathering an even greater breadth of GP input, as well as comparing and contrasting those to the preferences of our patients and communities.

## Introduction

The physical setting can influence our mood and how we perceive the social situation. It can also determine our likelihood of interacting with others, and also influences the form that interaction will take and how long it is likely to last ^1^. General practitioner’s (GP’s) consulting rooms differ from country to country, not only in language and cultural influences, but also in architectural design and placement of exam tables, desks, and chairs. Communication proves integral for GPs ^2^. The incorporation of computers and electronic medical records has further changed the physical environment and the way communication occurs. A GP consultation is one of the central experiences of the patient-physician interaction with both the location of the GP’s desk and the furniture arrangement impacting that encounter ^3^.

A clinical encounter requires a doctor, a patient, and two chairs at a minimum. Additionally, most practices will also have an office desk as well as a computer. These elements (two chairs and a desk) can be placed in various arrangements. Dannenberg & Burpee argue that the design focusing solely on safety regulations, such as following building codes and avoiding fire hazards, lose all the possibilities of improving the doctor and patient experience ^4^. Though these safety aspects are extremely important, engineers should not forego consideration of the medical encounter itself. For example, the physician’s desk can compartmentalize the physician and patient in opposite spaces - effectively serving as a “barrier” and interfering with communication ^5^. Furthermore, the power dynamics relationship (how power affects a relationship between two or more people) can be directly affected by the GP’s desk position, including the computer ^6, 7^ and furniture arrangement that can negatively influence the physician-patient relationship. In some cases, the introduction of the computer in the consultation room has led patients and physicians to make comparisons with the past and the “two players” consultation where computers - the third party - was not present ^8^. In one study that included a focus group, patients preferred to sit beside the physician with a clear view of the computer screen ^9^.

The question of where the GP should sit to promote optimal patient-centered care was raised in a Google Group discussion of the World Organization of Family Doctors (WONCA) Rural Working Party. After several email exchanges, the authors decided to develop a questionnaire-based survey. The aim of this study was to explore and investigate the layout of GP’s consulting rooms around the world and to describe differences encountered.

## Methods

After a literature review, a specific questionnaire exploring this topic was not found and the authors decided to develop one. This was an exploratory questionnaire intended to examine the possible layouts of consultation rooms within practices.

Between July 3rd and August 2nd 2018, an internet-based questionnaire on Google Docs ( *Extended data*) was distributed by email and WhatsApp to several worldwide rural medicine social media and WhatsApp groups as this allowed reaching a varied number of countries. The communities form a part of the WONCA Rural Working Party network and contain approximately 1200 GPs and trainees.

**Figure 1.**
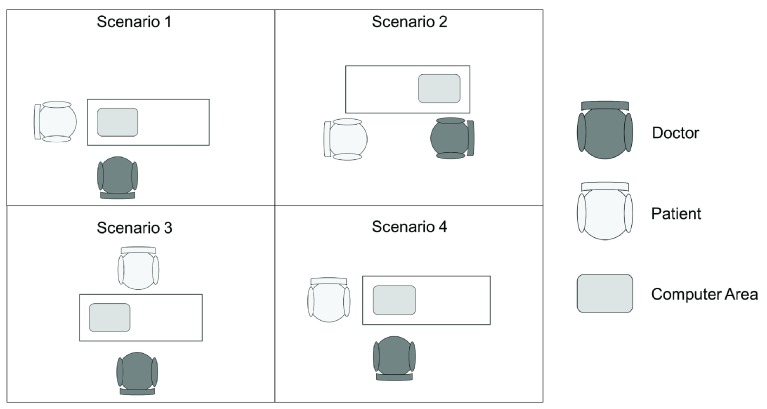
Proposed scenarios of practice with a computer. Participants could opt to describe their own practice if none of the scenarios matched.

The questionnaire was designed with three distinct sections. First, the GP demographic profile was collected including gender, year of graduation from medical school, country of graduation, type of practice (private or public), if they were in a rural/remote or urban area, and if there was a computer in their practice. The second part included questions relating to the office layout ( Figure 1). We proposed four scenarios as suggested in the Google group discussions. If the practice design was not represented in the scenarios proposed, the participants had the option to describe their own set up. Finally, there was a section for questionnaire feedback. This included a free text section and a question about satisfaction of the questionnaire (ranked from extremely dissatisfied to extremely satisfied).

The four scenarios proposed with a computer were ( Figure 1):

- Scenario 1: GP and patient with a 90° angle facing the desk with the computer on the corner.
- Scenario 2: GP and patient directly facing each other with the computer at the desk.
- Scenario 3: GP and patient separated by a desk with a computer in the middle/corner
- Scenario 4: GP and patient with a 90° angle facing each other with the computer on the corner.
- Other scenarios: Describe your own consulting room design

We analyzed the results of the study questionnaire using Chi-squared tests. The results were considered significant with p<0.05. The statistical programs Epi Info v7.2.2.1 and SPSS v23 were used for the statistical analyses. We did not analyze the qualitative responses in this study as they were intended to improve the design of a future questionnaire.

## Results

In total, 502 responses were collected during the 30-day period the questionnaire was active; 328 of the respondents (65.3%) were women and 174 (34.7%) were men. The country of practice with the most respondents was Romania with 199 responses (39.6%), followed by Spain with 140 (27.9%), Brazil with 51 (10.2%), Mexico with 32 (6.4%) and India with 16 (3.2%). We asked respondents to describe their practice area as rural/remote or urban; 114 respondents described their area as rural/remote (22.7%) and 379 as urban (75.5%). A total of 296 respondents described their practice as public (59%) and 184 as private (36.7%).

Regarding computers at the practice, 482 out of 502 (96%) respondents had a computer at their practice and 20 (4%) did not. All except one of the respondents who didn’t have a computer were from developing countries - mainly Nigeria (40%), India (15%), and Brazil (15%).

We analyzed the countries of practice in four main geographical regions: Europe, Asia-Pacific, the Americas (North and South America), and Africa.

In relation to practice layout, the most common setup in Europe was scenario 3, with the doctor and the patient separated by a desk (66%). This is also the most widely used layout in the Americas (46%), closely followed by scenario 4, with the GP and the patient with a 90° angle facing each other (41%). Scenario 4 was also the most common layout in Asia-Pacific (80%) and Africa (56%).

When comparing geographical regions, scenario 3 was used significantly more often in Europe than in the Americas (p=0.0003), whereas scenario 4 is used significantly more often (p<0.0005) in the Americas than in Europe. We did not analyze differences in the layouts between Asia-Pacific and Africa as we had so few responses. Layouts of practice by geographical are reported in Table 1.

**Table 1.** Layout of practice by geographical area.

			Scenario				Total
			1	2	3	4	
Zone	Europe	Count	20	65	232	33	350
		% within Zone	6%	19%	66%	9%	100%
	Asia-Pacific	Count	0	1	0	4	5
		% within Zone	0%	20%	0%	80%	100%
	America	Count	3	9	41	37	90
		% within Zone	3%	10%	46%	41%	100%
	Africa	Count	3	1	0	5	9
		% within Zone	33%	11%	0%	56%	100%
Total		Count	26	76	273	79	454
		% within Zone	6%	17%	60%	17%	100%

There were no statistically significant differences in the layout of the practice depending upon whether the practice was rural or urban (scenario 1, p=0.411; scenario 2, p=0.627; scenario 3, p=0.953; scenario 4, p=0.360).

We analyzed responses according to the respondents’ year of medical school graduation and established three cohorts: before 1990, between 1990 and 2010 and after 2010. Amongst those who graduated before 1990, the most common scenario was number 3 (54%), followed by scenario 2 (27%). Amongst those who graduated between 1990 and 2010, the most common scenario was number 3 (71 **%**). Amongst those who graduated after 2010, the most common layout was scenario 4 (55%), followed by scenario 3 (31%). Scenario 2 was used significantly more often by respondents who graduated before 1990 than by respondents who graduated between 1990 and 2010 (p=0.003) and those who graduated after 2010 (p=0.003). Scenario 3 was used significantly more often by respondents who graduated between 1990 and 2010 than respondents who graduated before 1990 (p=0.0002) and those who graduated after 2010 (p<0.0005). Finally, Scenario 4 was used significantly more often by respondents who graduated after 2010 than by respondents who graduated before 1990 (p=0.0002) and respondents who graduated between 1990 and 2010 (p=0.0002). Layouts of practice by year of graduation are reported in Table 2.

**Table 2.** Practice layout by year of graduation of the GP.

			Scenario				Total
			1	2	3	4	
Graduation	<1990	Count	13	52	104	25	194
		% within grad3	7%	27%	54%	13%	100%
	1990–2010	Count	14	21	153	27	215
		% within grad3	7%	10%	71%	13%	100%
	>2010	Count	3	6	20	36	65
		% within grad3	5%	9%	31%	55%	100%
Total		Count	30	79	277	88	474
		% within grad3	6%	17%	58%	19%	100%

Most female GPs reported using scenario 3 (66%), followed by scenario 2 (16%). Male GPs also used scenario 3 most often (44%), but scenario 4 (28%) was the second most popular layout. Scenario 1 was used statistically significantly more often by male rather than female respondents (p=0.01), while scenario 3 was used statistically significantly more often by female than male respondents (p<0.0005). In contrast, scenario 4 was used more often by male than female respondents (p=0.0002). Layouts of practice by gender are reported in Table 3.

**Table 3.** Exam room layout by gender of GP.

			Scenario				Total
			1	2	3	4	
Gender	Female	Count	14	51	207	44	316
		% within Gender	4%	16%	66%	14%	100%
	Male	Count	16	28	69	44	157
		% within Gender	10%	18%	44%	28%	100%
Total		Count	30	79	277	88	474
		% within Gender	6%	17%	58%	19%	100%

Finally, the participants rated the questionnaire with 104 (20.9%) extremely satisfied, 187 (38.6%) moderately satisfied, 185 (37.1%) neither satisfied or nor dissatisfied, 16 (3.2%) moderately dissatisfied and 6 (1.2%) extremely dissatisfied. In addition, the participants’ main feedback of how to improve the questionnaire was to allow participants to design their own consultation room and/or to have picture illustrations.

## Discussion

**Figure 2.**
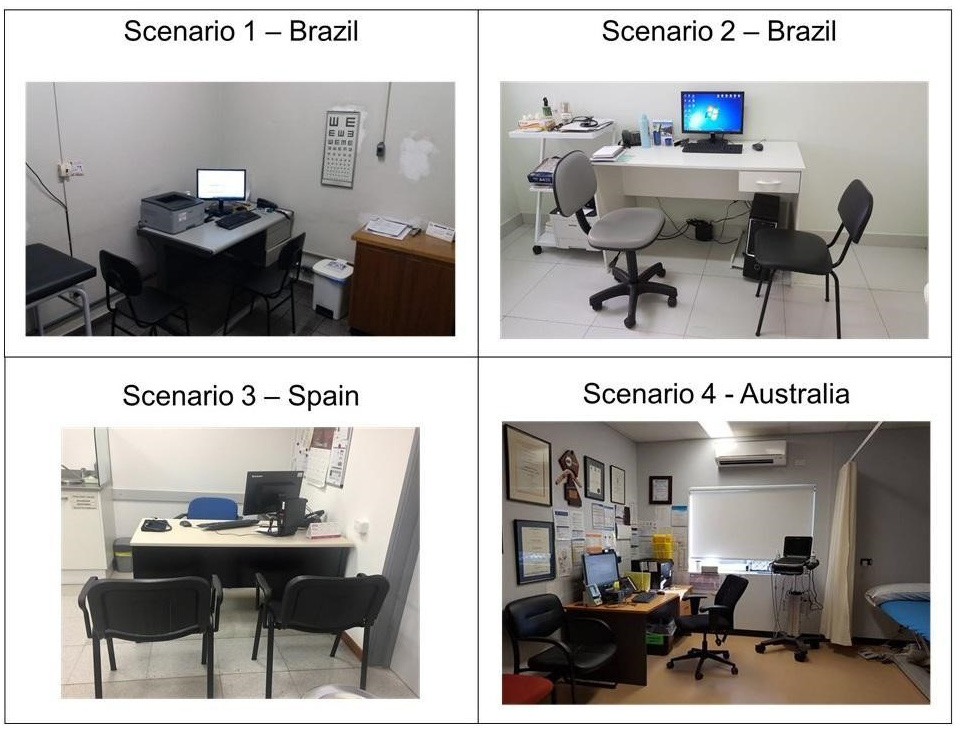
Real examples of practice layouts. All images were authorized for use by the general PR actioners who sent in the pictures.

This study found that the position of the GP’s desk differs internationally, within countries, and according to year of graduation as well as the gender of the GP. Each approach has a social and cultural context ( Figure 2).

The position of the chairs, computer and desk can directly affect the doctor-patient relationship ^3, 6^. Jacobs has previously described that arranging chairs perpendicularly (e.g. Scenario 1) implies teamwork and support, chairs placed side-by-side augments education and instruction, while chairs arranged face-to-face (e.g. Scenario 2) show expertise and can be used to gain compliance ^10^. Scenario 3 was the setup most widely used in Europe. Because of the computer interference and the table in between, this layout does not necessarily support ‘expertise’ of the physician while simultaneously distancing the patient ^10^.

In this study, women preferred scenario 3 (66%), followed by scenario 2 (16%). Generally, female GPs reassure and encourage patients more compared to their male counterparts ^2^ who most often used scenario 3 (44%) - noting that scenario 4 (28%) was the second most popular. The choice of a more “protective” layout with the desk separating physician and patient could be a gender-related as GPs are increasingly facing violence, harassment, and threatening behavior ^11^.

Computers can increase the accuracy of the patient record and enhance physician efficiency but can also negatively interfere with the physician-patient interaction **.** The majority of GP’s (96%) in this study reported having a computer in the room. Frankel *et al.* listed the possible aspects in which a computer may interfere with a consultation: organization of the visit, verbal and nonverbal behavior, computer navigation and spatial navigation of the consulting room. Frankel also stated that “computering” could affect communication, which is the most powerful instrument available to the physician ^12^. Sinsky and Beasley compared the risk of texting while driving to the risk of “texting while doctoring”; physicians should not practice “computer-centered medicine” ^13^. Therefore, consulting rooms must be designed so that the computer is not the center of care but so that it is convenient and suited to the ergonomics of the physician. The multi-tasking involved in using a computer and the flow of the patient-physician-computer interaction can be complex and stressful ^12^. A study conducted in Belfast examining the content of consultations with a significant psychological component showed that when the patient had a psychological complaint, physicians focused more on the patient rather than the computer and balanced their use of the computer and communication ^14^. Moreover, it has been shown that changing the layout of a consultation room has the potential to improve communications between patients and doctors ^15^. Clinicians who have more barriers integrating the computer in the consulting room could have more difficulties dealing with multi-tasking and this could have a negative effect on the physician-patient relationship ^12, 16^.

The patient experience is constructed consciously and unconsciously, and design affects patient perception ^5, 10^. Why is design layout in Europe different from the Americas? Why is scenario 3 preferred in Europe but scenario 4 preferred in the Americas? Although there is no definitive answer for these questions, it is interesting to reflect on the learning matrix from different countries and experiences.

Also note the dynamic reflected by the year of graduation. For those who graduated before 1990, the table between the patient and general practitioner is preferred (54%), and this increases to 70% for graduates between 1990–2010, and then decreases to 55% for younger GPs (graduating after 2010) who prefer scenario 4. This may reflect the emphasis on a more patient-centered approach the authors have noticed in recent years.

It has been suggested that consultation spaces need to be more adaptable so that additional family members can be included when necessary ^10^ and - when using the computer - preferably the patient should be able to see the computer screen ^17^. However, the need to understand each community and cultural competency are linked to the consulting room design. Modifying current consulting room designs should open-up new opportunities of interaction between the GP and patients.

This study has several limitations. First, the number of scenarios was limited and it was difficult to determine patient and GP locations from participants’ descriptions. This cohort of respondents represents those who have access to the Internet or knowledge of this survey - noting limited numbers of respondents from Africa, North America, and Northern Europe. The results were concentrated in some countries and continents more than others where a broader representation would likely offer better data. Furthermore, other factors such as different designs to those in the proposed scenarios, satisfaction of doctors and patients in the chosen design for the consulting room and cultural differences were not covered by this research could influence consulting room design. The studies that exist have focused mainly on the developed world experience and we were unable to expand the scope much into developing countries. Lastly, the patient’s viewpoint and preferences were not taken into consideration. Nevertheless, this study serves as an interesting starting point for further investigations to understand consulting room design worldwide and reflect upon it.

## Conclusions

The position of the patient’s chair differs between and within countries in addition to distinctions based on gender and year of graduation. Modifying current consulting room designs according to patient’s and doctor’s preferences could open up new opportunities for interaction between GPs and their patients, while continuing to remain conscious of both community and cultural differences during planning. Next steps should focus on gathering an even greater breadth of GP input as well as comparing and contrasting those to the preferences of our patients and communities.

## Ethical approval

The study protocol has been approved by the Catalan Institute in Primary Care Research (IDIAP Jordi Gol) Health Care Ethics Committee (code P18/202). Prior to starting the questionnaire, participants read the following: “Dear Participant: This pilot questionnaire aims to collect data for a study about consulting room from all around the world in Family Medicine/General Practice offices. The participation and engagement in this questionnaire is free, voluntary and anonymous. Thank you for participating. -Research Team.” Participant consent was therefore obtained if the participant continued with the questionnaire.

To ensure confidentiality, all data has been anonymized.

## Data availability

### Underlying data

Open Science Framework: Where is the Doctor’s Chair, https://doi.org/10.17605/OSF.IO/9FWU7 ^18^.

This project contains the following underlying data:

- Spreadsheet containing anonymized questionnaire responses

### Extended data

Open Science Framework: Where is the Doctor’s Chair, https://doi.org/10.17605/OSF.IO/9FWU7 ^18^.

This project contains the following extended data:

- Questionnaire

Data are available under the terms of the Creative Commons Zero “No rights reserved” data waiver (CC0 1.0 Public domain dedication).
