# Supplementary material for: Where is the patient’s chair? Differences in general practitioner consultation room layouts - an exploratory questionnaire
Source: F1000Res. 2020 Apr 17;8:1439. Originally published 2019 Aug 15. [Version 2] doi: 10.12688/f1000research.19565.2 (PMC7194339; doi:10.12688/f1000research.19565.2)
Supplement: Peer review history

# Peer review: Reviewer response for version 2

**DOI:** 10.5256/f1000research.25024.r62480

**Published:** 2020-04-27

**Authors:** Miguel-Angel Mayer

**Affiliations:** 1 Research Programme on Biomedical Informatics (GRIB), Hospital del Mar Medical Research Institute (IMIM), Pompeu Fabra University, Barcelona, Spain; 2 Information and Communication Technologies Working Group, Family Physicians Association of Catalonia (CAMFiC), Barcelona, Spain

The revisions made are appropriate based on the the doubts and suggestions included in the first review. I have no further comments to make. The paper is scientifically valid in its current form.

Is the work clearly and accurately presented and does it cite the current literature?

Partly

If applicable, is the statistical analysis and its interpretation appropriate?

Yes

Are all the source data underlying the results available to ensure full reproducibility?

Yes

Is the study design appropriate and is the work technically sound?

Yes

Are the conclusions drawn adequately supported by the results?

Partly

Are sufficient details of methods and analysis provided to allow replication by others?

Partly

Reviewer Expertise:

Electronic Health Records, Comorbidities, Quality of clinical data, Participatory health, Social media

I confirm that I have read this submission and believe that I have an appropriate level of expertise to confirm that it is of an acceptable scientific standard.

# Peer review: Reviewer response for version 2

**DOI:** 10.5256/f1000research.25024.r62479

**Published:** 2020-04-17

**Authors:** Ferdinando Petrazzuoli

**Affiliations:** 1 Center for Primary Health Care Research, Clinical Research Center, Lund University, Lund, Sweden

The authors have addressed appropriately the concerns I raised in my previous peer review report. The manuscript is very interesting and  now ready for immediate indexing.

Is the work clearly and accurately presented and does it cite the current literature?

Partly

If applicable, is the statistical analysis and its interpretation appropriate?

Yes

Are all the source data underlying the results available to ensure full reproducibility?

Yes

Is the study design appropriate and is the work technically sound?

Yes

Are the conclusions drawn adequately supported by the results?

Yes

Are sufficient details of methods and analysis provided to allow replication by others?

Yes

Reviewer Expertise:

dementia, communication skills

I confirm that I have read this submission and believe that I have an appropriate level of expertise to confirm that it is of an acceptable scientific standard.

# Peer review: Reviewer response for version 1

**DOI:** 10.5256/f1000research.21451.r58086

**Published:** 2020-02-05

**Authors:** Miguel-Angel Mayer

**Affiliations:** 1 Research Programme on Biomedical Informatics (GRIB), Hospital del Mar Medical Research Institute (IMIM), Pompeu Fabra University, Barcelona, Spain; 2 Information and Communication Technologies Working Group, Family Physicians Association of Catalonia (CAMFiC), Barcelona, Spain

- The manuscript presents a very interesting subject: the current general practitioner consultation room layouts in several countries. The study is based on an internet-based questionnaire.
- There are some aspects that require some clarifications. For instance, the concept of "health clinic design" is not clear enough. It seems advisable to use another terminology such as "consultation room design" or "healthcare settings design".
- The authors mention that the aim of this study was also to describe any significant differences among consulting rooms around the world; what do “significant differences” mean? In terms of? Please specify.
- In the introduction, what does “the power relationship” sentence mean? It should be clarified.
- In the methods section the sentence “A specific questionnaire exploring this topic was not found” is stated. How did the authors arrive to this conclusion?
- Please, review this sentence: “there was a section for questionnaire feedback in order to allow for future improvements.”
- There is some information missing in the methods and results sections: the sentence “distributed by email to several worldwide rural medicine social media groups” is mentioned, please specify in which social media groups or platforms they were distributed or the criteria for selecting them, the number of questions, the fact that among them 296 respondents described their practice as public and 184 as private and that means there were some respondents missing. The number of countries that participated in the study, why are Asia and Australia are included in the same group? Apart from the graduation year, the age of participants is not included, and it would have been of interest to have a more complete respondents’ profile.
- In the discussion section the authors mention that “The position of the chairs and desk can directly affect the doctor-patient relationship”. This statement should be supported by several references.
- In the conclusion section we read that “Modifying current consulting room designs opens up new opportunities for interaction between GPs and their patients…” What kind of modifications are the authors referring to and in which of the scenarios described? What kind of opportunities? It seems that it is of great interest to describe these points even briefly.

Is the work clearly and accurately presented and does it cite the current literature?

Partly

If applicable, is the statistical analysis and its interpretation appropriate?

Yes

Are all the source data underlying the results available to ensure full reproducibility?

Yes

Is the study design appropriate and is the work technically sound?

Yes

Are the conclusions drawn adequately supported by the results?

Partly

Are sufficient details of methods and analysis provided to allow replication by others?

Partly

Reviewer Expertise:

Electronic Health Records, Comorbidities, Quality of clinical data, Participatory health, Social media

I confirm that I have read this submission and believe that I have an appropriate level of expertise to confirm that it is of an acceptable scientific standard, however I have significant reservations, as outlined above.

# Peer review: Reviewer response for version 1

**DOI:** 10.5256/f1000research.21451.r55281

**Published:** 2019-11-15

**Authors:** Ferdinando Petrazzuoli

**Affiliations:** 1 Center for Primary Health Care Research, Clinical Research Center, Lund University, Lund, Sweden

This manuscript addresses an interesting topic: how orientation and seating arrangements may influence the doctor patient’s relationship. The manuscript is interesting but a bit poor in terms of references. The topic has a lot to do with personal communication and I strongly advice to introduce the concepts and the references illustrated below.

 Orientation refers to body angles adopted when people talk, such as directly facing or shoulder to shoulder. It concerns the position of the trunk, rather the head, and it marks the degree of intimacy in the conversation and the levels of friendship. ^1^

 It is useful to look at proximity and orientation together since it has been found that there can be an inverse relationship between them that is, direct face to face alignment is linked to greater interpersonal distance and sideways angling to closer distance. ^1^ This would be expected in situations where orientation was being used to compensate for excessive closeness. ^2^ Orientation can also be used to include or exclude others from the group during discussion. ^1^

 Early studies of seating behaviour by Sommer ^3^ in North America, replicated by Cook ^4^ in the UK, point to some interesting differences in seating arrangements if individuals are given a choice of where to sit when involved in different sorts of activities.

 Environmental factors are also important. The physical setting can influence our mood and how we perceive the social situation. It can also determine our likelihood of interacting with others, and also influences the form that interaction will take and how long it is likely to last. ^1^ Hall distinguished between fixed-feature and semi-fixed feature elements of the environment. ^5^ The former includes everything that is relatively permanent or not easily modifiable, such as the architectural layout of a house, size and shape of rooms, and the material used in their construction while semifixed features are much easier to move around or modify, and include furniture, lighting, temperature. According to Knapp and Hall based on such characteristics, we form impressions of our surroundings organized around 6 dimensions. ^6^ These are *:*

- ***Formality: concerns cues leading to decisions about how casual in what is said and done or if a more ritualized or stylized performance is demanded.***
- ***Warmth: here one feels comfortable, secure, and at ease in what are regarded as convivial surroundings.***
- ***Privacy: has to do with the extent to which interactors feel that they have the space to themselves or whether others may intrude or eavesdrop.***
- ***Familiarity: involving impressions of having encountered this type of setting before and knowing how to deal with it (or not as the case maybe).***
- ***Constraint: concerns perceptions of how easy it is to enter and leave the situation.***
- ***Distance: address how close, either physically or psychologically, we feel to those with whom we share the space.***

 

 These perceptions shape the types of interactions we engage in and how we experience them. ^1^ The way in which work space is arranged and utilised sends strong signals about the status and authority of occupants, the kinds of tasks and activities being implicitly proposed, and last but not least the desirability and appropriateness of focused communication in that situation. ^1^

Is the work clearly and accurately presented and does it cite the current literature?

Partly

If applicable, is the statistical analysis and its interpretation appropriate?

Yes

Are all the source data underlying the results available to ensure full reproducibility?

Yes

Is the study design appropriate and is the work technically sound?

Yes

Are the conclusions drawn adequately supported by the results?

Yes

Are sufficient details of methods and analysis provided to allow replication by others?

Yes

Reviewer Expertise:

dementia, communication skills

I confirm that I have read this submission and believe that I have an appropriate level of expertise to confirm that it is of an acceptable scientific standard, however I have significant reservations, as outlined above.

## References

[1] : Skilled Interpersonal Communication.2016; 10.4324/9781315741901 10.4324/9781315741901

[2] : An Empirical Comparison of Three Theories of Nonverbal Immediacy Exchange. *Human Communication Research*.1998;24(4) : 10.1111/j.1468-2958.1998.tb00429.x 501-535 10.1111/j.1468-2958.1998.tb00429.x

[3] : Personal Space: The Behavioral Basis of Design. *Prentice-Hall*.1969;

[4] : Experiments on Orientation and Proxemics. *Human Relations*.1970;23(1) : 10.1177/001872677002300107 61-76 10.1177/001872677002300107

[5] : The Hidden Dimension. *Garden City, N.Y.: Doubleday*.1966;

[6] : Nonverbal communication in human interaction. *Cengage Learning*.2013;
